# Watchful Waiting in Pediatric Acute Otitis Media: A Real Practice Approach or an Intangible Desideratum?

**DOI:** 10.3390/medicina59030520

**Published:** 2023-03-07

**Authors:** Elena-Lia Spoială, Iuliana Magdalena Stârcea, Ileana Katerina Ioniuc, Romică Sebastian Cozma, Daniela Carmen Rusu, Laura Bozomitu, Vasile Valeriu Lupu, Codruţa Olimpiada Iliescu Haliţchi, Vasile Eduard Roşu, Solange Tamara Roşu, Cristina Gavrilovici

**Affiliations:** 1 Pediatrics Department, “Grigore T. Popa” University of Medicine and Pharmacy, 700115 Iasi, Romania; 2 ENT Department, “Grigore T. Popa” University of Medicine and Pharmacy Iasi, Universitatii Street 16, 700115 Iasi, Romania; 3 Nursing Departmentsolange, “Grigore T. Popa” University of Medicine and Pharmacy, 700115 Iasi, Romania

**Keywords:** acute otitis media, watchful waiting, children, antibiotics

## Abstract

Acute otitis media (AOM) in children is one of the leading causes of health care visits and antibiotic prescriptions worldwide. The overall aim of the current study is twofold: 1. to analyze and discuss the antibiotic prescription patterns in AOM in children without complications or risk factors and 2. to assess to what extent the watchful-waiting approach is a real practice or a mere desideratum. We performed an electronic search in the PubMed and Embase databases from 2013 to 2023 to capture original research studies investigating antibiotic prescribing patterns for AOM in children. Among the 12 papers included in the analysis, the antibiotic prescription rate ranged from 44.8% to 98%. Our study reveals similarities regarding the use of amoxicillin as a first-line antibiotic in pediatric AOM, but also discrepancies in the watchful-waiting approach attitude and in the choice of second or third-line antimicrobial agents. The proportion of cases managed with the watchful-waiting approach ranged from 7.5% (Australia) to 55.2% (Finland). Denmark was the only country reporting penicillin V as a first-choice regimen for children with AOM, which fulfils the guidelines’ recommendations. The most unsatisfying rate of amoxicillin use was recorded in Japan, contrary to the recommendations of local guidelines. The use of quinolones was reported in two out of twelve studies, with the highest proportion in Japan, where tosufloxacin was used in 21.4% of the total number of cases. The duration of the antibiotic regimens was analyzed in three out of twelve papers. Since global antibiotic overuse contributes to the emergence of antibiotic resistant bacteria, new strategies are needed to increase the rate of watchful waiting and to promote the judicious use of antibiotics.

## 1. Introduction

Currently, antibiotic overuse is still an increasing threat towards bacterial resistance, leading to avoidable short- and long-lasting side effects for the population, such as higher medical costs, prolonged hospital stays, and increased mortality [1]. Acute otitis media (AOM) is one of the leading causes of antibiotic prescription in children [2]. The main etiology of AOM, even after the introduction of pneumococcal vaccination, is represented by Streptococcus pneumoniae, Haemophilus influenzae, Moraxella catarrhalis, Streptococcus pyogenes, and Staphylococcus aureus [3]. Less frequent pathogens include Gram negative bacilli such as Escherichia coli, Pseudomonas aeruginosa, or Salmonella [4,5]. However, AOM is not a pure bacterial disease: respiratory syncytial virus, rhinovirus, adenovirus, coronavirus, bocavirus, influenza virus, parainfluenza virus, enterovirus, and human metapneumovirus were recently identified in children with AOM [6]. In the last decades, AOM management has received much attention as, even in the post-pneumococcal conjugate vaccine era, it was estimated that 23% of children experience at least one episode of AOM up to the age of 1 year old and 60% of them have multiple episodes [7].

A meta-analysis performed by Venekamp et al. [8] revealed that the use of antibiotics in AOM has no early analgesic effect and that 78% of cases spontaneously remit without complications after 10 to 12 days. Nowadays, the preferred strategy is the watchful-waiting approach in children older than 24 months, whereas, for younger children, most of the guidelines recommend immediate antibiotic treatment [9]. According to the American Academy of Pediatrics, antibiotic therapy should be reserved for AOM cases with severe manifestations (a fever of 39 °C or higher or moderate or severe otalgia or otalgia for at least 48 h) [10]. Once the administration of an antibiotic regimen is decided, high-dose amoxicillin should be the first-line choice, followed by amoxicillin/clavulanic acid in case of treatment failure [10].

The overall aim of the current study is twofold: 1. to analyze and discuss the antibiotic prescription patterns in AOM in children without complications or risk factors and 2. to assess to what extent the watchful-waiting approach is a real practice or a mere desideratum.

## 2. Materials and Methods

We performed an electronic search in the PubMed and Embase databases from 2013 to 2023, to capture original research studies investigating antibiotic prescribing patterns for acute otitis media in children. We used the following search terms: [“acute otitis media”] AND [(“treatment”) OR (“therapy”) OR (“management”) OR (“antibiotics”) OR (“watchful-waiting” OR “wait and see”)] AND [(“children”) OR (“pediatric”) OR (“paediatric”)]. Our results were limited to humans and restricted to the English and French language. All of the significant articles were manually searched to further identify any additional eligible studies. We focused only on original studies reporting the approach to antibiotic treatment and/or watchful waiting in nonrecurrent and uncomplicated AOM in children (0–18 years old) without comorbidities which might influence the decision of antibiotherapy.

## 3. Results

**Figure 1:**
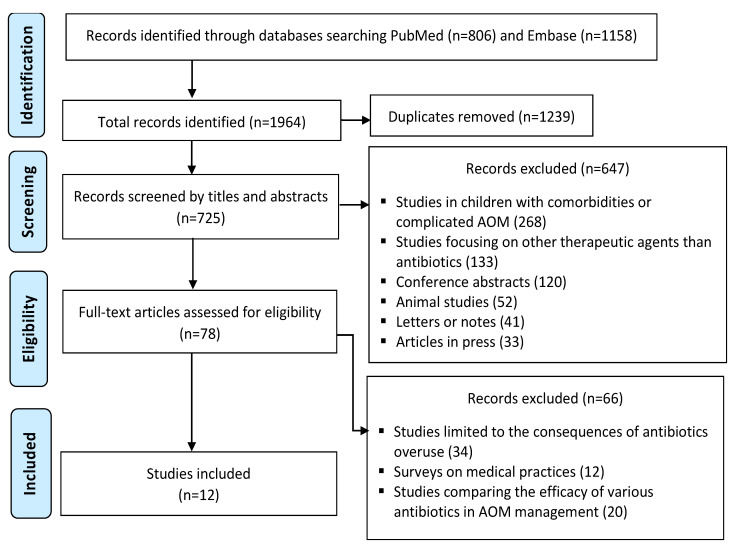
Prisma flow-chart.

The literature search retrieved 1964 references (Figure 1). After removing 1239 duplicates, 725 titles and abstracts were screened. From the latter, 647 were further excluded due to the lack of eligibility criteria (studies focusing on other therapeutic agents than antibiotics—e.g., analgesic agents, corticosteroids, intravenous immunoglobulin, antihistaminic agents; studies in children with recurrent or complicated AOM; studies in children with comorbidities which might influence the choice of antibiotic treatment—e.g., upper respiratory tract infections, pneumonia, gastroenteritis, urinary tract infections; animal studies; manuscripts in a language other than English or French; or irrelevant research papers for our purpose: conference abstracts, letters or notes, articles in press). After reading 78 full text articles, 66 were excluded as being limited to the consequences of antibiotics overuse, or surveys on medical practices, or comparisons regarding the efficacy of various antibiotics in AOM management. In total, 12 papers were included in this review.

The main characteristics of the included studies are summarized in Table 1. We focused on the population size and age, the antibiotic prescription rate and the preferred regimen (including treatment duration if available), and the attitude towards a watchful-waiting approach. In addition, other valuable concluding remarks which were difficult to be compared between the studies were provided in the last column of the paper. Although the population size lacks homogeneity, ranging from 278 to 25,212,264 children, the selected papers assess the preferred antimicrobial therapy worldwide, in countries from Europe, North America, Asia, and Australia. The antibiotic choices and trends, the attitude towards watchful waiting and the adherence to the guidelines will be detailed in the following sections.

### 3.1. Antibiotic Choice

The antibiotic prescription rate ranged from 44.8% to 98%. Seven out of the twelve papers included in this analysis report amoxicillin as the first-choice antibiotic throughout the study period. The highest rate of amoxicillin use in an analysis of the antibiotic prescription patterns in children aged 2 years or older was 81.2% [19]. Amoxicillin/clavulanic acid was the preferred antibiotic agent in the two studies reporting AOM cases from Italy: Palma et al. [18] described a 51% rate of amoxicillin/clavulanic acid use, whereas Barbieri et al. [15] described a rate of 36%. Denmark was the only country reporting penicillin V as a first-choice regimen for children with AOM [22].

Cephalosporins were the third most used antibiotic for AOM after amoxicillin and amoxicillin/clavulanic acid in five out of twelve studies. Specific antibiotics belonging to the first three generations of cephalosporins were preferred in studies from Australia (cephalexin) [21], Spain (cefuroxime) [20], France and Italy (cefpodoxime proxetil) [15,17,18], and the United States (cefdinir) [19]. Seven out of twelve papers reported the use of macrolides in a small proportion of cases, ranging from 1.3% to 10%. Marom et al. [13] and Frost et al. [19] reported that azithromycin was the preferred macrolide in population samples with AOM from Israel and the United States.

The use of quinolones was reported in only two out of twelve studies, with the highest proportion reported by Yamaguchi et al. in Japan, where tosufloxacin was used in 21.4% of the total number of cases included and in 21.5% at the end of the 5-year period (in 2018) [16]. In Australia, Balasundaram et al. reported that the third most used antibiotic for AOM was ciprofloxacin (in 7% of cases), being situated in frequency between aminopenicillins and cephalosporins [21]. The use of carbapenemes was also reported by Yamaguchi et al. in Japan, in 4% of cases analyzed throughout the study period (2014 to 2018) and in 3.5% of cases in 2018 [16].

Only three of the twelve papers [19,20,21] analyzed the duration of the antibiotic regimens. Compared to the studies from Colorado [19] and Aragon [20], where the most used duration was 10 days and 7 days, respectively, in the study from Australia [21], the majority of the children (59.3%) received a 5-day antibiotic course.

### 3.2. Antibiotic Trends

Five of the twelve studies depict the trends in time regarding antibiotic treatment in AOM, bringing valuable information about the changes in preferred therapeutic agents in France, Finland, Italy, Korea, and Japan. The studies reveal an improvement in the use of amoxicillin and amoxicillin/clavulanic acid, with the most striking increasing rate of amoxicillin use reported by Levy et al. in France (2.6% in 2009 versus 66.1% in 2012) [17]. Yamaguchi et al. reported only a slight increase in amoxicillin use in Japan, from 24% throughout the study (2012 to 2018) period to 28.9% in 2018 [16]. Although analyzing a substantial population sample of more than 25 million children, the report from Korea does not separate amoxicillin from amoxicillin/clavulanic acid and reports a slight increase of 5.7% in the use of both antimicrobials in 2018 compared to 2012 [11]. However, the use of cephalosporins decreased, from 3.3% at the end of a 7-year period in the study from Korea [11] to 28.4% at the end of a 4-year period in the study from France [17]. In Italy, no significant improvement in the antibiotic prescription rate and in amoxicillin use was observed after the introduction of the guidelines [18].

### 3.3. Watchful-Waiting Approach

The watchful-waiting approach was mentioned in nine of the twelve studies. The proportion of cases managed with watchful waiting ranged from 7.5% [21] to 55.2% [14]. Moreover, two of the twelve papers provide an insight into the outcomes of these children: among the children who benefited from watchful waiting, antibiotic treatment was required in only 2.8% of cases in the study by Smolinski et al. [12] and in 53.5% of cases in the study by García Ventura et al. [20]. The clinician’s specialty may influence the decision regarding immediate antibiotherapy. In the study by Smolinski et al. [12], the watchful-waiting approach was more frequently adopted by otolaryngologists (odds ratio (OR) 5.45, 95% CI 5.21–5.70) compared to pediatricians or general practitioners, whereas in the study by Yamaguchi et al. [12], physicians in hospitals were more likely to prescribe amoxicillin than those in otolaryngology clinics (OR 1.71, 95% CI 1.63–1.79). In Spain, García Ventura et al. [20] reported that children younger than 2 years old were less likely to benefit from a watchful-waiting approach (36%) compared to children aged 6 to 14 years old (63.4%), In contrast, the report from Finland [14] showed that only 44.1% of antibiotic prescriptions were in children younger than 2 years old.

### 3.4. Adherence to Guidelines

In order to provide an accurate insight into medical practices regarding the best choice of antibiotic regimen in AOM, we further analyzed the local guidelines (Table 2) as geographical peculiarities around antimicrobial resistance might trigger differences in antibiotic recommendations.

All the guidelines analyzed in Table 2 encourage the watchful-waiting approach in children with mild and uncomplicated AOM. The earliest age for considering the “wait-and-see” strategy is 2 years old in six out of eleven guidelines and 6 months in four out of eleven guidelines, whereas the guidelines from Finland [25] do not mention a specific threshold of age but encourage the watchful-waiting approach in nonpurulent AOM. Ten out of eleven guidelines recommend the use of amoxicillin, whereas cephalosporins and macrolides are reserved for special situations, such as allergy to first-line treatment or treatment failure. The fluoroquinolones are not included in the recommended group of antibiotics in any of the guidelines. After analyzing the guidelines’ recommendations, the following observations can be made:

- The high rate of cephalosporins use reported by Park et al. in Korea [11] is not justified by the guidelines, as this class of antibiotics is reserved only for children with non-type I hypersensitivity. Furthermore, macrolides are recommended only for children with a history of type I hypersensitivity reaction to penicillin (e.g., urticaria, anaphylaxis). The fact that 64% of the children with AOM receiving antibiotics were 0–23 months old was expected as immediate antibiotherapy is required in all the children <6 months old with AOM.
- Amoxicillin was the most frequent choice of antibiotic by the clinicians in both studies reporting AOM cases from the United States [12,19]. However, in most of the cases (54.1%) reported by Frost et al. [12], using a 10-day regimen of antibiotics exceeded the duration recommended by the guidelines.
- In the reports from Spain [20] and Denmark [22], the first-choice antibiotic was decided according to the local guidelines. Moreover, the regimen duration reported in Spain [16] respected the indications of the local guidelines.
- The report from Israel [11] revealed the highest rate of azithromycin use, which is recommended by the local guidelines only in cases of major penicillin allergy. As the cephalosporins do not seem to be recommended at all, 48%–54% of AOM episodes were inappropriately treated, against the guideline recommendations. The results are similar to those reported by the Australian report, as 39% of children received inappropriate antibiotic treatment according to the Australian guidelines, including a 7% rate of ciprofloxacin use.
- The study from Italy [15] reported a lack of adherence to the guidelines in cases of treatment failure, as instead of switching from amoxicillin to amoxicillin/clavulanic acid, the most frequent antibiotic switches were from amoxicillin directly to cephalosporins.
- The most unsatisfying rate of amoxicillin use was recorded in Japan [16], contrary to the recommendations in the guidelines. Moreover, both quinolones and carbapenemes are not recommended by the local guidelines. The use of tosufloxacin in 21.4% of cases was also unexpected.
- In the study by Levy et al. (France), an increasing rate of amoxicillin use was observed after the introduction of the 2011 French guidelines [17].
- The lowest proportion of antibiotic prescriptions in children <2 years old was reported by Csonka et al. in Finland, which is contrary to our expectations, as children under 6 months old are recommended to undergo immediate antibiotherapy according to ten out of eleven guidelines [14].

## 4. Discussion

Currently, the most encouraged strategy in the guidelines on the management of AOM is watchful waiting, while the use of antibiotics has only limited indications [9]. According to the American Academy of Pediatrics, the first-line antibiotic choice is represented by amoxicillin, whereas an additional β lactamase inhibitor may be useful in concurrent conjunctivitis, history of recurrent AOM, or recent treatment with amoxicillin in the last 30 days [10]. Due to its excellent middle ear penetration, low cost, high tolerability, and narrow antimicrobial spectrum, an adequate dosage of Amoxicillin can be effective even when ear cultures reveal in vitro resistance against *Streptococcus pneumoniae*, beta-lactamase-negative *Haemophilus influenzae*, and *Streptococcus pyogenes* [32].

In Romania, we have analyzed the medical records of 3103 pediatric patients aged 2–18 years admitted and diagnosed with AOM over a 10-year period (from July 2010 to December 2021) in a tertiary hospital. Overall, clinicians recommended immediate antibiotic treatment in 98.03% of cases. The preferred antimicrobial agent used in 42.12% of cases was a third-generation cephalosporin (ceftriaxone), followed by a second-generation cephalosporin (cefuroxime) in 15.79% of cases. Amoxicillin was recommended in only 1.16% of cases, whereas amoxicillin/clavulanate was used in only 3.93% of cases. In this context, it is not surprising that worrying proportions of multidrug-*resistant Streptococcus pneumoniae* strains (82.85%), as well as high resistance rates to trimethoprim/sulfamethoxazole, macrolides, and lincosamides were reported in the same hospital [33].

Antibiotic treatment in AOM in children is prescribed accordingly to the most common microorganisms isolated from the middle ear fluids. The spectrum of activity should cover, at least, *Streptococcus pneumoniae* and *Haemophilus influenzae*. Amoxicillin, although widely recommended, is not totally harmless. The use of high-dose amoxicillin had the second-highest rates of diarrhea (13.8%) and the highest rates of a generalized rash (6.5%) in a meta-analysis including 82 papers [34]. However, it still remains the best choice for having the highest activity of the commonly prescribed oral agents against the most common bacterial AOM pathogens [35]. The fluoroquinolones have a good coverage of the main bacteria involved in AOM. The Pediatric Food and Drug Administration indications include the use of ciprofloxacin for chronic suppurative otitis media or malignant otitis externa caused by *Pseudomonas aeruginosa* [36]. Levofloxacin was also efficacious for the treatment of recurrent otitis media, but with no additional benefit when compared to amoxicillin/clavulanic acid [37]. Approved for pediatric AOM in Japan since 2010, tosufloxacin exhibits potent activity against nontypeable *Haemophylus influenzae* [38]. However, quinolones should be kept as a last resort [39], due to serious potential side effects, such as the prolongation of the corrected QT interval, phototoxicity, liver enzyme abnormalities, arthropathy, and cartilage and tendon toxicity [40]. Furthermore, besides the short-term effects, which are usually manageable and include allergic reactions, nausea, vomiting, diarrhea, and rash [34], recent research reveals worrying data about the long-term effects. In a cohort of children aged 2–10 years old including a large sibling-control analysis, Wernroth et al. (2020) found a significant association between antibiotics prescriptions in the first 12 months of life, primarily for AOM or respiratory tract infections, and an increased risk of type 1 diabetes (adjusted hazard ratio (HR) 1.19 (95% CI 1.05–1.36)) [41].

Our analysis reveals that further improvements are needed in antibiotic stewardship in AOM in children. The studies included in our review are incontestable proof that effective strategies for guideline implementation are needed in order to encourage the watchful-waiting approach and to provide suitable antibiotic treatment, by avoiding broad-spectrum antibiotics. Lautenbach et al. [42] reported that the use of an electronic algorithm to identify inappropriate antibiotic prescribing for AOM in children was highly accurate, showing that 97% of antibiotic prescriptions were justifiable, but only 9% of cases received an appropriate antibiotic.

The barriers for adopting guideline recommendations may be due to a lack of guideline knowledge and current practice inertia, but also due to parental pressure and the fear of severe complications [43]. In studies comparing the outcomes of children receiving immediate antibiotherapy versus watchful waiting, no serious AOM-related consequences were reported in either group, but with the cost of increasing adverse effects and a negative impact on quality of life in cases managed with immediate antibiotherapy [44,45]. In a large cohort of 30 ,159 children, broad-spectrum treatment was not associated with a lower rate of treatment failure (3.4% for broad-spectrum antibiotics vs 3.1% for narrow-spectrum antibiotics) [46].

The watchful-waiting approach is highly encouraged nowadays in uncomplicated cases of AOM [47], as in healthy children, the innate immunity is able to manage the AOM episodes without any complications or sequelae [48]. Recent updates to AOM guidelines attempt to reduce antibiotic overuse even more. For instance, based on the results of a randomized controlled trial comprising children from England and Wales [49], the National Institute for Health and Care Excellence (NICE) guidelines on AOM agreed on using eardrops containing an anesthetic and an analgesic as a first-line treatment in order to reduce antibiotic consumption in children who did not need immediate antibiotics [50]. This recommendation is also supported by the conclusions of a five-trial systematic review of 391 participants: the anesthetic drops did provide better pain relief than the inactive drops [51]. However, the reduction in antibiotic consumption following the prescription of ear drops in AOM requires validation in larger studies. Otic drops with a combined anesthetic and analgesic formula (antipyrine and benzocaine) were removed in 2015 in the United States, due to insufficient evidence of their safety and effectiveness, although a topical anesthetic is still available [52]. Moreover, preventive strategies may also contribute to the reduction in antibiotic overuse in AOM. As obesity was associated with a higher risk of developing AOM [53], recent studies have investigated the role of the diet in AOM and concluded that the traditional Mediterranean diet may prevent recurrent AOM in children [54]. However, in pediatrics, the investigations are rarely conducted on large samples for ethical reasons and the validity of these results may be questionable [55].

## 5. Conclusions

The judicious use of antibiotics may decrease the danger of high antibiotic resistance rates. Clinicians should be encouraged to delay antibiotics prescriptions and to overcome the fear of rare complications in children with AOM. Although important improvements have been made in the stewardship of antibiotics in AOM, increased efforts are still needed to improve the adherence to guidelines.

Our study reveals similarities regarding the use of amoxicillin as a first-line antibiotic in pediatric AOM, but also discrepancies in the watchful-waiting approach attitude and in the choice of second or third-line antimicrobial agents. Among the 12 papers included in the analysis, the antibiotic prescription rate ranged from 44.8% to 98%. Denmark was the only country reporting penicillin V as a first-choice regimen for children with AOM, which fulfils the guidelines’ recommendations. The most unsatisfying rate of amoxicillin use was recorded in Japan, contrary to the guidelines’ recommendations. The use of quinolones was reported in only two of the twelve studies, with the highest proportion in Japan, where tosufloxacin was used in 21.4% of the total number of cases. The proportion of cases managed with the watchful-waiting approach ranged from 7.5% (Australia) to 55.2% (Finland). Since global antibiotic overuse contributes to the emergence of antibiotic resistant bacteria, new strategies are needed to increase the rate of watchful waiting and to promote the judicious use of antibiotics.

## Figures and Tables

**Table 1:** Summary of the basic characteristics of the included studies.

Study, Publication Year, Region	Period	Population Sample	Antibiotic Prescription Rate	Prescribed Antibiotics	Attitude Towards Watchful-Waiting Approach	Observations
Park et al., 2021, Korea [11]	7 years (2012 to 2018)	25,212,264 children aged 0–6 years old	Not specified	AMX/AMC (56.1% in 2012; 61.8% in 2018) cephalosporins (35.1% in 2012; 31.8% in 2018) macrolides (8.7% in 2012; 6.4% in 2018)	Not specified	Of the children with AOM receiving antibiotics, 64% were 0–23 months old.
Smolinski et al., 2022, United States [12]	15 years (2005 to 2019)	2,176,617 children aged 1–12 years old	77.8%	AMX (52.6%) AMC (15%) cephalosporins (21.4%) macrolides (10%) sulfonamides (0.9%)	Among 22.2% of cases managed with WW ***, only 2.8% cases required antibiotic treatment (days 4 to 7).	Otolaryngologists adopted more frequently the WW approach (odds ratio 5.45, 95% CI 5.21–5.70) compared to pediatricians.
Marom et al., 2021, Israel [13]	8 years (2011 to 2018)	491,106 children aged 0–10 years old	71.5%	AMX (75.6%) AMC (13.8%) azithromycin (8%)	28.5% of cases managed with WW.	Azithromycin was the most cited antibiotic in cases with treatment failure (6.6%).
Csonka et al., 2022, Finland [14]	7 years (2014 to 2020)	357,390 children aged 0–18 years old	44.8%	AMX (19.3% in 2014; 18.1% in 2020) AMC (12.6% in 2014; 13.2% in 2020) macrolides (7.5% in 2014; 3.5% in 2020)	55.2% of cases managed with WW.	Pediatricians prescribed AMX or AMC to 80% of cases compared to 67% by GPs and 55% by ENT physicians. The lowest proportion (44.1%) of antibiotic prescriptions was in children <2 years old.
Barbieri et al., 2021, Italy [15]	7 years (2012 to 2018)	157,915 children aged 0–14 years old	Not specified	AMC (36%) AMX (30%) cephalosporins (30%) macrolides (4%)	Not specified	The most common antibiotic switches due to treatment failure were from AMC to cephalosporins (16.7%), followed by switches from AMX to AMC (9.6%).
Yamaguchi et al., 2021, Japan [16]	5 years (2014 to 2018)	109,051 children aged 0–7 years old	Not specified	AMX (24% throughout the study period; 28.9% in 2018) cephalosporins (32.6% throughout the study period; 28.5% in 2018) Tosufloxacin (21.4% throughout the study period; 21.5% in 2018 carbapenemes (4% throughout the study period; 3.5% in 2018) macrolides (6.4% throughout the study period; 5.7% in 2018)	Not specified	Although there was an increasing trend in the use of AMX, the rate was still low. Physicians in hospitals were more likely to prescribe AMX than those in otolaryngology clinics (aOR = 1.71, 95% CI 1.63 to 1.79).
Levy et al., 2014, France [17]	4 years (2009 to 2012)	14,661 children aged 0.5–2 years old	85.1%	AMX * (2.6% in 2009; 66.1% in 2012) AMC ** (62% in 2009; 27.7% in 2012) Cefpodoxime proxetil (33.6% in 2009; 5.2% in 2012)	14.9% of cases managed with WW.	The study highlights the importance of guidelines in decreasing the prescription of broad-spectrum antibiotics as AMX became the most frequently prescribed antibiotic for AOM in 2012, after the introduction of the 2011 French guidelines.
Palma et al., 2015, Italy [18]	7 years (2007 to 2013)	4573 children aged 0–14 years old	81%	AMC (51%) AMX (32%) Cefuroxime axetil (11%) macrolides (2%)	19% of cases managed with WW.	The introduction of the Italian guidelines had no significant effect on the frequency of the antibiotic prescribing rate (82% versus 81%). Of the antibiotics prescribed, 48% were in children under 2 years old.
Frost et al., 2020, Colorado (United States) [19]	1 year (2018)	1025 children aged 2–18 years old	98%	AMX (81.2%) AMC (18.8%) cefdinir (5.5%) azithromycin (1.3%)	2% of cases managed with WW.	Children aged 2 to 5 years old or admitted to emergency departments received ≥10 days of antibiotics compared with those admitted to pediatric clinics (*p* < 0.001).
García Ventura et al., 2021, Aragon (Spain) [20]	6 years (2013 to 2018)	696 children aged 0–14 years old	81.3%	AMX (74.6%) AMC (17.0%) topical medication (3.49%) cefuroxime (3.01%)	Among 18.7% of cases managed with WW, 53.5% cases required antibiotic treatment.	Children aged 0 to 2 years old were treated more frequently with immediate antibiotics (63%) compared to children aged 6 to 14 years old (36.6%).
Balasundaram et al., 2019, Southeast Queensland (Australia) [21]	1 year (January 2016 to June 2017)	305 children aged 0–15 years old	62%	AMX (63%) AMC (18%) ciprofloxacin (7%) cephalexin (4%)	7.5% of cases managed with WW.	According to the Australian guidelines, 39% of children received inappropriate antibiotic treatment.
Olsen et al., 2020, Denmark [22]	A 4-week winter period in 2017/2018	278 children aged 0–7 years old	74%	penicillin V (60%) AMX/AMC (22%) macrolides (1.9%)	26% of cases managed with WW.	Fever, otorrhea, and poor general condition were associated with immediate antibiotic treatment.

* AMX—amoxicillin; AMC **—amoxicillin/clavulanate; WW ***—watchful waiting.

**Table 2:** Antimicrobial therapy and watchful-waiting recommendations according to regional guidelines.

Region	Recommendations According to the Local Guidelines			Watchful-Waiting Approach
	First-Line Antibiotic	Second-Line Antibiotic/Treatment Failure	Third Line/Allergy to First Line	
Korea [23]	AMX, 5–7 days for mild cases, 10 days for moderate and severe cases	AMC, 5–7 days for mild cases, 10 days for moderate and severe cases	Ceftriaxone	WW in children >6 months old and reevaluation after 2–3 days in cases of mild AOM in children with no recent history of taking antibiotics and no other accompanying disease.
United States [10]	AMX, <2 years of age: 10 days; 2–5 years of age: 7 days; >6 years of age: 5–7 days	AMC	Cefdinir OR ceftriaxone	WW in children >6 months old in the absence of severe signs or symptoms (mild otalgia for less than 48 h, temperature <39 °C).
Colorado (United States) [10]				
Israel [24]	AMX	AMC OR cefuroxime	Azithromycin OR clarithromycin	WW for 1–2 days in non-severe cases in children ≥6 months old who can be followed up by a primary care physician.
Finland [25]	AMX or AMC, 5–7 days	Ceftriaxone	Cefaclor or macrolide	WW in nonpurulent AOM and reexamination after 2–3 days if symptoms continue.
Italy [26]	AMX, 5–10 days	AMC, 5–10 days	Macrolide	WW in children >2 years old and reevaluation after 2–3 days in cases of mild AOM (unilateral, without otorrhea).
Japan [27]	AMX, 5 days	AMC, 5 days	Cefditoren pivoxil	WW for 3 days in mild (based on a severity score) AOM.
France [28]	AMX, 8–10 days	AMC OR ceftriaxone, 3 days	Erythromycin-sulfafurazole/ cotrimoxazole/ cefpodoxime	WW in children >2 years old and reevaluation after 2–3 days in cases of mild AOM (no high fever or severe otalgia).
Aragon (Spain) [29]	AMX, 5 days	AMC OR ceftriaxone, 7–10 days	Cefuroxime	WW in children >2 years old and reevaluation after 2 days in cases of mild AOM (fever <39 °C, light pain).
Southeast Queensland (Australia) [30]	AMX, 7–14 days	High doses of AMX (90 mg/kg)	Cefuroxime	WW in children >6 months old and reevaluation after 2 days in cases of mild AOM (if the child is not immunocompromised or of Aboriginal descent or from the Torres Strait Islands).
Denmark [31]	Penicillin V, 7 days	AMC, 7 days	Clarithromycin	WW in children >2 years old and reevaluation after 3 days in the absence of otorrhea or other severe symptoms.

## Data Availability

Not applicable.
